# *N*-Hydr­oxy-*N*-*o*-tolyl­acetamide

**DOI:** 10.1107/S1600536808002468

**Published:** 2008-01-30

**Authors:** Yu-Hao Li, Rui Liu, Xiang-Ning Zhang, Hong-Jun Zhu

**Affiliations:** a Department of Applied Chemistry, College of Science, Nanjing University of Technology, Nanjing 210009, People’s Republic of China; b Jiangsu Pesticide Research Institute Co. Ltd., Nanjing 210019, People’s Republic of China

## Abstract

In the mol­ecule of the title compound, C_9_H_11_NO_2_, the methyl C atom bonded to the ring and the N atom lie in the benzene ring plane. An intra­molecular O—H⋯O hydrogen bond results in the formation of a five-membered planar ring, which is oriented at a dihedral angle of 81.37 (3)° with respect to the benzene ring. In the crystal structure, inter­molecular O—H⋯O hydrogen bonds link the mol­ecules stacked along the *b* axis. There are also π–π inter­actions between benzene rings with a face-to-face stacking distance of 3.434 Å.

## Related literature

For related literature, see: Fu *et al.* (2000 ▶). For bond-length data, see: Allen *et al.* (1987 ▶).
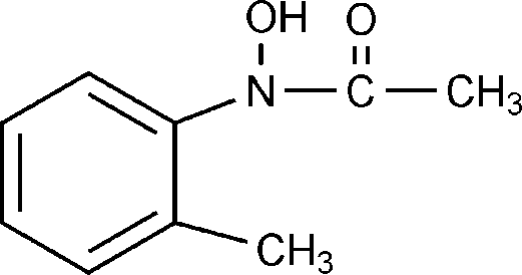


## Experimental

### 

#### Crystal data

C_9_H_11_NO_2_
*M* *_r_* = 165.19
Monoclinic, 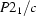

*a* = 7.7890 (16) Å
*b* = 7.1570 (14) Å
*c* = 15.613 (3) Å
β = 96.86 (3)°
*V* = 864.1 (3) Å^3^
*Z* = 4
Mo *K*α radiation
μ = 0.09 mm^−1^
*T* = 294 (2) K
0.40 × 0.30 × 0.30 mm

#### Data collection

Enraf–Nonius CAD-4 diffractometer
Absorption correction: ψ scan (North *et al.*, 1968 ▶) *T*_min_ = 0.965, *T*_max_ = 0.973
1821 measured reflections
1695 independent reflections
1187 reflections with *I* > 2σ(*I*)
*R*_int_ = 0.054
3 standard reflections frequency: 120 min intensity decay: none

#### Refinement

*R*[*F* ^2^ > 2σ(*F* ^2^)] = 0.067
*wR*(*F* ^2^) = 0.182
*S* = 1.05
1695 reflections
109 parameters
H-atom parameters constrained
Δρ_max_ = 0.30 e Å^−3^
Δρ_min_ = −0.35 e Å^−3^

### 

Data collection: *CAD-4 Software* (Enraf–Nonius, 1985 ▶); cell refinement: *CAD-4 Software*; data reduction: *XCAD4* (Harms & Wocadlo, 1995 ▶); program(s) used to solve structure: *SHELXS97* (Sheldrick, 2008 ▶); program(s) used to refine structure: *SHELXL97* (Sheldrick, 2008 ▶); molecular graphics: *PLATON* (Spek, 2003 ▶); software used to prepare material for publication: *SHELXTL* (Sheldrick, 2008 ▶).

## Supplementary Material

Crystal structure: contains datablocks I, global. DOI: 10.1107/S1600536808002468/hk2420sup1.cif

Structure factors: contains datablocks I. DOI: 10.1107/S1600536808002468/hk2420Isup2.hkl

Additional supplementary materials:  crystallographic information; 3D view; checkCIF report

## Figures and Tables

**Table 1:** Hydrogen-bond geometry (Å, °)

*D*—H⋯*A*	*D*—H	H⋯*A*	*D*⋯*A*	*D*—H⋯*A*
O1—H1*A*⋯O2	0.82	2.19	2.608 (3)	112
O1—H1*A*⋯O2^i^	0.82	1.97	2.719 (3)	152

Symmetry code: (i) 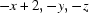.

**Fig. 1.**
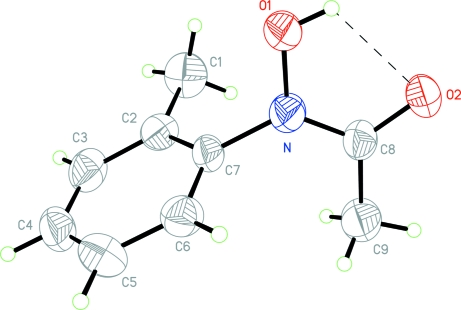
The molecular structure of the title molecule, with the atom-numbering scheme. Displacement ellipsoids are drawn at the 50% probability level.

**Fig. 2.**
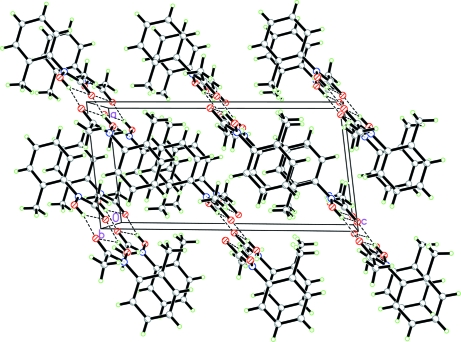
A packing diagram of (I). Hydrogen bonds are shown as dashed lines.

## supplementary crystallographic information

### Comment

The title compound, (I), contains hydroxy and formyl groups, which can react with different groups to prepare various function organic compounds. It is a kind of aromatic organic intermediate that can be used for many fields such as pesticide, paper making *etc*. (Fu *et al.*, 2000). We report herein its crystal structure.

In the molecule of (I), (Fig. 1), the bond lengths (Allen *et al.*, 1987) and angles are within normal ranges. The atoms N and C1 lie in the benzene ring plane. The intramolecular O—H···O hydrogen bond (Table 1) results in the formation of a five-membered planar ring A (O1/H1A/O2/N/C8). The dihedral angle between five- and six-membered rings is 81.37 (3)°.

In the crystal structure, intermolecular O—H···O hydrogen bonds (Table 1) link the molecules stacked along the *b* axis (Fig. 2), in which they may be effective in the stabilization of the structure.

There are also the π-π interactions of benzene rings with a face-to-face stacking distance of 3.434 Å.

### Experimental

The title compound, (I), was prepared by the literature method (Fu *et al.*, 2000). Crystals suitable for X-ray analysis were obtained by dissolving (I) (0.5 g) in petroleum ether (20 ml) and evaporating the solvent slowly at room temperature for about 7 d.

### Refinement

H atoms were positioned geometrically, with O—H = 0.82 Å (for OH) and C—H = 0.93 and 0.96 Å for aromatic and methyl H, respectively, and constrained to ride on their parent atoms, with *U*_iso_(H) = *xU*_eq_(C,*O*), where *x* = 1.2 for aromatic H, and *x* = 1.5 for all other H atoms.

### Crystal data

**Table:**

C_9_H_11_NO_2_	*F*_000_ = 352
*M**_r_* = 165.19	*D*_x_ = 1.270 Mg m^−^^3^
Monoclinic, *P*2_1_/*c*	Melting point: 350 K
Hall symbol: -P 2ybc	Mo *K*α radiation λ = 0.71073 Å
*a* = 7.7890 (16) Å	Cell parameters from 25 reflections
*b* = 7.1570 (14) Å	θ = 10–13º
*c* = 15.613 (3) Å	µ = 0.09 mm^−^^1^
β = 96.86 (3)º	*T* = 294 (2) K
*V* = 864.1 (3) Å^3^	Block, colorless
*Z* = 4	0.40 × 0.30 × 0.30 mm

### Data collection

**Table:**

Enraf–Nonius CAD-4 diffractometer	*R*_int_ = 0.054
Radiation source: fine-focus sealed tube	θ_max_ = 26.0º
Monochromator: graphite	θ_min_ = 2.6º
*T* = 294(2) K	*h* = −9→9
ω/2θ scans	*k* = 0→8
Absorption correction: ψ scan(North *et al.*, 1968)	*l* = 0→19
*T*_min_ = 0.965, *T*_max_ = 0.973	3 standard reflections
1821 measured reflections	every 120 min
1695 independent reflections	intensity decay: none
1187 reflections with *I* > 2σ(*I*)	

### Refinement

**Table:**

Refinement on *F*^2^	Secondary atom site location: difference Fourier map
Least-squares matrix: full	Hydrogen site location: inferred from neighbouring sites
*R*[*F*^2^ > 2σ(*F*^2^)] = 0.067	H-atom parameters constrained
*wR*(*F*^2^) = 0.182	*w* = 1/[σ^2^(*F*_o_^2^) + (0.05*P*)^2^ + 1.5*P*] where *P* = (*F*_o_^2^ + 2*F*_c_^2^)/3
*S* = 1.05	(Δ/σ)_max_ < 0.001
1695 reflections	Δρ_max_ = 0.31 e Å^−^^3^
109 parameters	Δρ_min_ = −0.35 e Å^−^^3^
Primary atom site location: structure-invariant direct methods	Extinction correction: none

### Special details

**Table:**

Geometry. All e.s.d.'s (except the e.s.d. in the dihedral angle between two l.s. planes) are estimated using the full covariance matrix. The cell e.s.d.'s are taken into account individually in the estimation of e.s.d.'s in distances, angles and torsion angles; correlations between e.s.d.'s in cell parameters are only used when they are defined by crystal symmetry. An approximate (isotropic) treatment of cell e.s.d.'s is used for estimating e.s.d.'s involving l.s. planes.
Refinement. Refinement of *F*^2^ against ALL reflections. The weighted *R*-factor *wR* and goodness of fit *S* are based on *F*^2^, conventional *R*-factors *R* are based on *F*, with *F* set to zero for negative *F*^2^. The threshold expression of *F*^2^ > σ(*F*^2^) is used only for calculating *R*-factors(gt) *etc*. and is not relevant to the choice of reflections for refinement. *R*-factors based on *F*^2^ are statistically about twice as large as those based on *F*, and *R*- factors based on ALL data will be even larger.

### Fractional atomic coordinates and isotropic or equivalent isotropic displacement parameters (Å^2^)

**Table:**

	*x*	*y*	*z*	*U*_iso_*/*U*_eq_	
N	0.7518 (4)	0.1297 (4)	0.08788 (17)	0.0451 (7)	
O1	0.8117 (3)	−0.0536 (3)	0.08895 (15)	0.0554 (7)	
H1A	0.8888	−0.0624	0.0577	0.083*	
O2	0.9365 (3)	0.2046 (3)	−0.00399 (16)	0.0573 (7)	
C1	0.8476 (4)	0.1834 (6)	0.2703 (2)	0.0593 (10)	
H1B	0.8527	0.2003	0.3316	0.089*	
H1C	0.9065	0.2848	0.2461	0.089*	
H1D	0.9022	0.0675	0.2585	0.089*	
C2	0.6626 (4)	0.1799 (4)	0.23108 (19)	0.0406 (7)	
C3	0.5278 (5)	0.2025 (5)	0.2806 (2)	0.0508 (9)	
H3A	0.5533	0.2200	0.3398	0.061*	
C4	0.3579 (5)	0.1999 (5)	0.2453 (2)	0.0536 (9)	
H4A	0.2706	0.2150	0.2805	0.064*	
C5	0.3164 (4)	0.1750 (5)	0.1577 (2)	0.0572 (9)	
H5A	0.2013	0.1721	0.1337	0.069*	
C6	0.4465 (4)	0.1545 (5)	0.1062 (2)	0.0454 (8)	
H6A	0.4194	0.1392	0.0470	0.054*	
C7	0.6189 (4)	0.1567 (4)	0.14250 (18)	0.0370 (7)	
C8	0.8214 (4)	0.2543 (5)	0.03929 (18)	0.0411 (7)	
C9	0.7578 (5)	0.4524 (5)	0.0400 (3)	0.0586 (10)	
H9A	0.8186	0.5270	0.0023	0.088*	
H9B	0.7779	0.5013	0.0975	0.088*	
H9C	0.6362	0.4555	0.0205	0.088*	

### Atomic displacement parameters (Å^2^)

**Table:**

	*U*^11^	*U*^22^	*U*^33^	*U*^12^	*U*^13^	*U*^23^
N	0.0584 (17)	0.0377 (14)	0.0420 (14)	0.0072 (12)	0.0179 (12)	0.0028 (12)
O1	0.0735 (16)	0.0420 (13)	0.0570 (15)	0.0174 (11)	0.0338 (12)	0.0085 (11)
O2	0.0654 (15)	0.0530 (15)	0.0599 (15)	0.0118 (12)	0.0343 (13)	0.0102 (12)
C1	0.054 (2)	0.066 (2)	0.057 (2)	−0.0089 (18)	0.0002 (16)	−0.0039 (18)
C2	0.0504 (18)	0.0360 (16)	0.0352 (16)	0.0026 (14)	0.0045 (13)	−0.0034 (12)
C3	0.073 (2)	0.0481 (19)	0.0339 (16)	0.0030 (17)	0.0155 (15)	−0.0029 (14)
C4	0.057 (2)	0.050 (2)	0.059 (2)	0.0045 (16)	0.0285 (17)	0.0015 (17)
C5	0.0411 (18)	0.064 (2)	0.066 (2)	0.0057 (17)	0.0044 (16)	−0.0010 (19)
C6	0.0486 (18)	0.0471 (19)	0.0401 (17)	0.0052 (15)	0.0043 (14)	0.0001 (14)
C7	0.0423 (16)	0.0363 (16)	0.0349 (15)	0.0036 (13)	0.0150 (12)	0.0012 (12)
C8	0.0440 (16)	0.0482 (18)	0.0318 (15)	0.0009 (14)	0.0076 (12)	0.0017 (13)
C9	0.069 (2)	0.044 (2)	0.066 (2)	0.0046 (17)	0.0243 (19)	0.0052 (17)

### Geometric parameters (Å, °)

**Table:**

N—C8	1.328 (4)	C3—H3A	0.9300
N—O1	1.392 (3)	C4—C5	1.378 (5)
N—C7	1.431 (4)	C4—H4A	0.9300
O1—H1A	0.8200	C5—C6	1.375 (5)
C1—C2	1.497 (5)	C5—H5A	0.9300
C1—H1B	0.9600	C6—C7	1.394 (4)
C1—H1C	0.9600	C6—H6A	0.9300
C1—H1D	0.9600	C8—C9	1.502 (5)
O2—C8	1.238 (3)	C9—H9A	0.9600
C2—C3	1.386 (4)	C9—H9B	0.9600
C2—C7	1.394 (4)	C9—H9C	0.9600
C3—C4	1.371 (5)		
			
C8—N—O1	118.8 (2)	C6—C5—C4	119.5 (3)
C8—N—C7	128.6 (3)	C6—C5—H5A	120.3
O1—N—C7	112.7 (2)	C4—C5—H5A	120.3
N—O1—H1A	109.5	C5—C6—C7	120.2 (3)
C2—C1—H1B	109.5	C5—C6—H6A	119.9
C2—C1—H1C	109.5	C7—C6—H6A	119.9
H1B—C1—H1C	109.5	C6—C7—C2	120.9 (3)
C2—C1—H1D	109.5	C6—C7—N	119.1 (3)
H1B—C1—H1D	109.5	C2—C7—N	119.9 (3)
H1C—C1—H1D	109.5	O2—C8—N	119.4 (3)
C3—C2—C7	117.1 (3)	O2—C8—C9	122.4 (3)
C3—C2—C1	121.7 (3)	N—C8—C9	118.2 (3)
C7—C2—C1	121.1 (3)	C8—C9—H9A	109.5
C4—C3—C2	122.2 (3)	C8—C9—H9B	109.5
C4—C3—H3A	118.9	H9A—C9—H9B	109.5
C2—C3—H3A	118.9	C8—C9—H9C	109.5
C3—C4—C5	120.1 (3)	H9A—C9—H9C	109.5
C3—C4—H4A	119.9	H9B—C9—H9C	109.5
C5—C4—H4A	119.9		
			
C7—C2—C3—C4	0.9 (5)	C1—C2—C7—N	2.0 (4)
C1—C2—C3—C4	179.9 (3)	C8—N—C7—C6	82.5 (4)
C2—C3—C4—C5	−0.3 (5)	O1—N—C7—C6	−98.0 (3)
C3—C4—C5—C6	−0.6 (6)	C8—N—C7—C2	−99.2 (4)
C4—C5—C6—C7	0.8 (5)	O1—N—C7—C2	80.4 (3)
C5—C6—C7—C2	−0.1 (5)	O1—N—C8—O2	0.4 (5)
C5—C6—C7—N	178.2 (3)	C7—N—C8—O2	179.9 (3)
C3—C2—C7—C6	−0.7 (4)	O1—N—C8—C9	−178.9 (3)
C1—C2—C7—C6	−179.7 (3)	C7—N—C8—C9	0.6 (5)
C3—C2—C7—N	−179.0 (3)		

### Hydrogen-bond geometry (Å, °)

**Table:**

*D*—H···*A*	*D*—H	H···*A*	*D*···*A*	*D*—H···*A*
O1—H1A···O2	0.82	2.19	2.608 (3)	112
O1—H1A···O2^i^	0.82	1.97	2.719 (3)	152

Symmetry codes: (i) −*x*+2, −*y*, −*z*.
